# Dammarane-Type 3,4-*seco*-Triterpenoid from Silver Birch (*Betula pendula* Roth) Buds Induces Melanoma Cell Death by Promotion of Apoptosis and Autophagy

**DOI:** 10.3390/molecules29174091

**Published:** 2024-08-29

**Authors:** Lukasz Szoka, Marcin Stocki, Valery Isidorov

**Affiliations:** 1 Department of Medicinal Chemistry, Medical University of Bialystok, 15-222 Białystok, Poland; 2 Institute of Forest Sciences, Białystok University of Technology, 15-351 Białystok, Poland; m.stocki@pb.edu.pl (M.S.); isidorov@uwb.edu.pl (V.I.)

**Keywords:** birch, dammarane, triterpenoid, apoptosis, autophagy

## Abstract

Despite unquestionable advances in therapy, melanoma is still characterized by a high mortality rate. For years, high expectations have been raised by compounds of natural origin as a component of pharmacotherapy, particularly by triterpenes found in the bark of birch trees. In this study, 3,4-*seco*-dammara-4(29),20(21),24(25)-trien-3-oic acid (SDT) was isolated from buds of silver birch and its mechanisms of cell death induction, including apoptosis and autophagy, were determined. Cytotoxicity of SDT was evaluated by the cell viability test and clonogenic assay, whereas induction of apoptosis and autophagy was determined by annexin V staining and Western blot. The results revealed dose- and time-dependent reductions in viability of melanoma cells. Treatment of cells for 48 h led to an increase in the percentage of annexin V-positive cells, activation of caspase-8, caspase-9, and caspase-3, and cleavage of PARP, confirming apoptosis. Simultaneously, it was found that SDT increased the level of autophagy marker LC3-II and initiator of autophagy beclin-1. Pretreatment of cells with caspase-3 inhibitor or autophagy inhibitor significantly reduced the cytotoxicity of SDT and revealed that both apoptosis and autophagy contribute to a decrease in cell viability. These findings suggest that 3,4-*seco*-dammaranes may become a promising group of natural compounds for searching for anti-melanoma agents.

## 1. Introduction

The medicinal effects of plants are integrally linked to the presence of pharmacologically active secondary metabolites. The largest and most structurally diverse chemical group of natural compounds is terpenes, and to date, more than 80,000 such compounds have been identified [1]. The linear precursor of triterpenes is the 30-carbon squalene, which undergoes cyclization after oxidation, resulting in over 100 different products containing multiple fused rings [2]. Subsequently, the basic triterpene structures undergo enzymatic modifications, in particular, oxygenation, alkylation, acylation, glycosylation, dehydrogenation, and formation of heterocyclic rings to yield more than 23,000 chemically diverse triterpenoids [3,4]. The pharmacological activity of triterpenes was extensively studied and anticancer [5], anti-inflammatory [6], antidiabetic [7], hepatoprotective [8], antiviral [9], and antibacterial [10] effects were reported.

One of the unusual reactions that triterpenoids undergo is the formation of *seco*-triterpenoids by opening a ring in the polycyclic triterpenoid scaffold. This can occur as early as at the stage of squalene cyclization or may be the result of oxidation of a carbon atom in the ring. In the second case, the reaction products are carboxylic acids or their esters [11,12]. *Seco*-triterpenoids show various activities, in particular cytotoxic, antiviral, antibacterial, and anti-inflammatory. Since parent structures are highly divergent, there is no general rule on how cleavage of the ring in triterpenoids affects their biological activity [12]. Considering the limited recognition of this natural group, investigation among *seco*-triterpenoids may become the way to obtain compounds with beneficial activity for human health, especially in cancer, which is a major challenge for healthcare. 

Melanoma was responsible for 325,000 new cases and 57,000 deaths in 2020 [13]. Cutaneous melanoma is typically managed with surgery and rarely with topical chemotherapy and radiotherapy, whereas systemic checkpoint immunotherapy and BRAF-targeted or MEK-targeted therapies are used in advanced melanoma. Since acquiring resistance is the major efficacy-limiting factor of targeted chemotherapy [14], there is an urgent need to continue investigation for new parental structures offering anti-melanoma activity. 

Silver birch (*Betula pendula* Roth) is a deciduous tree native to Europe and North Asia [15]. The species has pharmaceutical value, particularly bark, leaves, buds, and sap, which are used in treating renal and urinary tract diseases, rheumatism, and colds [16]. The triterpenoid fraction of silver birch bark is composed mainly by lupanes (betulin, lupeol, betulinic acid, betulone, betulonic aldehyde, lupenone, and betulonic acid) and oleananes (oleanolic acid, oleanolic aldehyde, and β-amyrin), while extracts from silver birch shoots also contain dammaranes (3,4-*seco*-dammara-4(29),20(21),24(25)-trien-3-oic acid, dammaradien-3-one, 20-hydroxy-3,4-*seco*-dammara-4(28),24-dien-3-oic acid, and cabraleone), ursanes (3,4-*seco*-urs-4(23),20(30)-dien-3-oic acid, 3,4-*seco*-urs-4(23),20(30)-dien-19-ol-3-oic acid) and other *seco*-triterpenoids (3,4-*seco*-olean-4(24)-en-19-on-3-oic acid) [17,18]. Triterpenes extracted from silver birch buds are betulonic acid, moronic acid, 3,4-*seco*-olean-4(24)-en-19-on-3-oic acid, 28-acetooxy-3,4-*seco*-olean-4(24),13(18)-diene-3-oic acid, 3,4-*seco*-dammara-4(29),20(21),24(25)-trien-3-oic acid, (20S)-hydroxy-3,4-*seco*-dammara-4(28),24-diene-3-oic acid, and (19R)-hydroxy-3,4-*seco*-taraxasta-4(24)-en-3-oic acid [19,20]. Notably, betulinic acid was identified as an effective anti-melanoma agent showing high selectivity towards cancer cells [21], while betulin scaffold is widely used for the design of novel compounds with improved anticancer activity [22,23]. 

**Figure 1:**
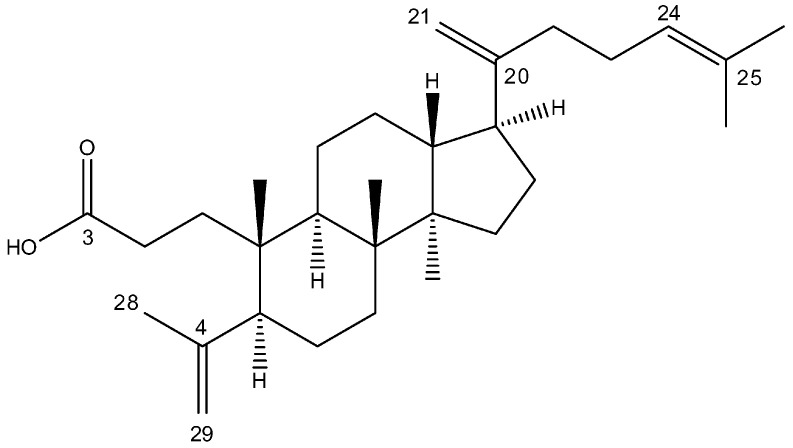
Chemical structure of 3,4-*seco*-dammara-4(29),20(21),24(25)-trien-3-oic acid (SDT).

The viability of cells treated with 3,4-*seco*-ursanes and 3,4-*seco*-oleananes isolated from downy birch buds was previously investigated. It was revealed that they moderately reduce the viability of colon cancer and gastric cancer cells by induction of apoptosis. Moreover, their toxicity in fibroblasts was lower compared to cancer cells [24]. Among 3,4-*seco*-dammaranes, dammarenolic acid and its derivatives showed potency against cervical cancer, leukemia, and melanoma cell lines [25,26]. In this study, 3,4-*seco*-dammara-4(29),20(21),24(25)-trien-3-oic acid (SDT) (Figure 1) was isolated from silver birch buds and its anticancer activity was investigated in melanoma cells.

## 2. Results

### 2.1. SDT Reduces Viability of Melanoma Cells

**Figure 2:**
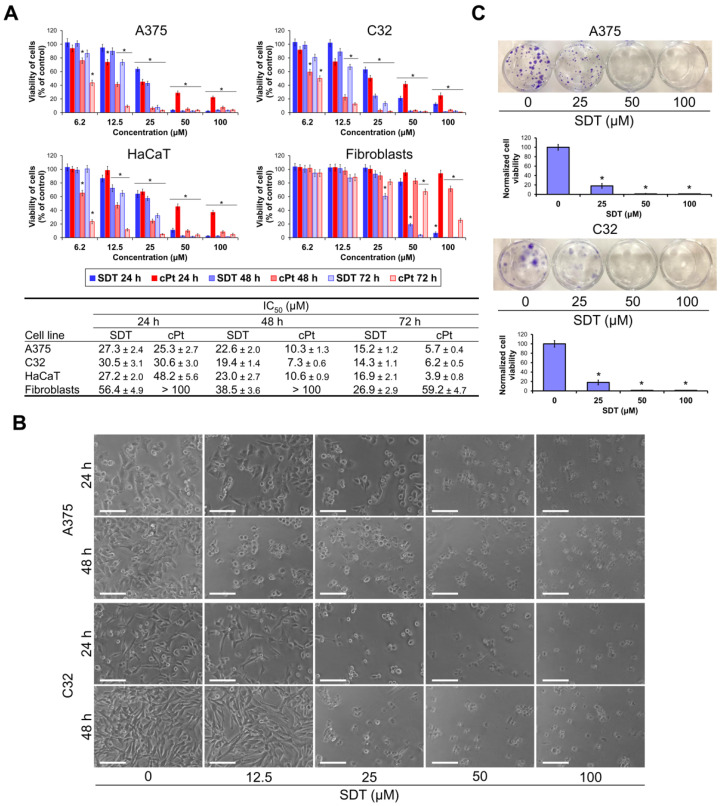
SDT decreases the viability of melanoma cells. (**A**) A375 and C32 melanoma cells, skin keratinocytes (HaCaT), and dermal fibroblasts were treated with different concentrations (6.2–100 μM) of SDT for 24, 48, and 72 h, and then cell viability was determined by MTT assay. Cisplatin (cPt) was used as a positive control. (**B**) Cell morphology of A375 and C32 melanoma cells treated with different concentrations (6.2–100 μM) of SDT for 24 and 48 h. Scale bar 100 μm, objective ×20. (**C**) Melanoma cell colonies were visualized using crystal violet staining following 7 days of cell treatment with SDT (25–100 μM) for 48 h. Data are presented as mean ± SD from three independent experiments. * *p* < 0.05 vs. control.

The cytotoxic activity of SDT in melanoma cell lines A375 and C32 was determined by an MTT assay. A well-known anticancer drug cisplatin (cPt) was used as the reference compound. Human normal dermal fibroblasts (CCD-25Sk) and skin keratinocytes (HaCaT) were used in order to assess the selectivity of the compounds. Cells were treated with SDT and cPt for 24, 48, and 72 h. As shown in Figure 2A, SDT showed a strong decrease in cell proliferation in a dose-dependent manner. The IC_50_ values of SDT in A375 and C32 after treatment for 24 h were 27.3 and 30.5 μM, respectively. These values decreased about twofold when exposure was prolonged to 72 h. The viability of fibroblasts was less affected by SDT and its IC_50_ value in all time points was twofold higher compared to melanoma cells. In contrast, IC_50_ values of SDT in keratinocytes were similar to those observed in A375 and C32 cells. The viability of melanoma cells incubated with cPt for 24 h was reduced to a similar extent as after treatment with SDT. However, cytotoxicity of cPt was strongly augmented with increasing time of the treatment reaching a fivefold decrease in IC_50_ values at 72 h time point. Fibroblasts were significantly less susceptible to the cytotoxic activity of cPt compared to melanoma cells. Its IC_50_ values after 48 and 72 h of incubation were tenfold higher than in A375 and C32 cells. Similarly, as in the case of SDT, the viability of keratinocytes treated with cPt was reduced almost identically compared to melanoma cells. As shown in Figure 2B, melanoma cells A375 and C32 incubated with 25 μM SDT showed loss of adhesion, cell rounding, and apoptotic body formation. Subsequently, a clonogenic assay was performed to determine the long-term effect of treatment with SDT (Figure 2C). Consistently, 25 μM SDT impaired colony formation by 80% in both melanoma cell lines. These data indicate that SDT effectively reduces melanoma cell viability and has modest selectivity to cancer cells when compared to fibroblasts.

### 2.2. SDT Induces Death of Melanoma Cells by Apoptosis and Non-Protective Autophagy

**Figure 3:**
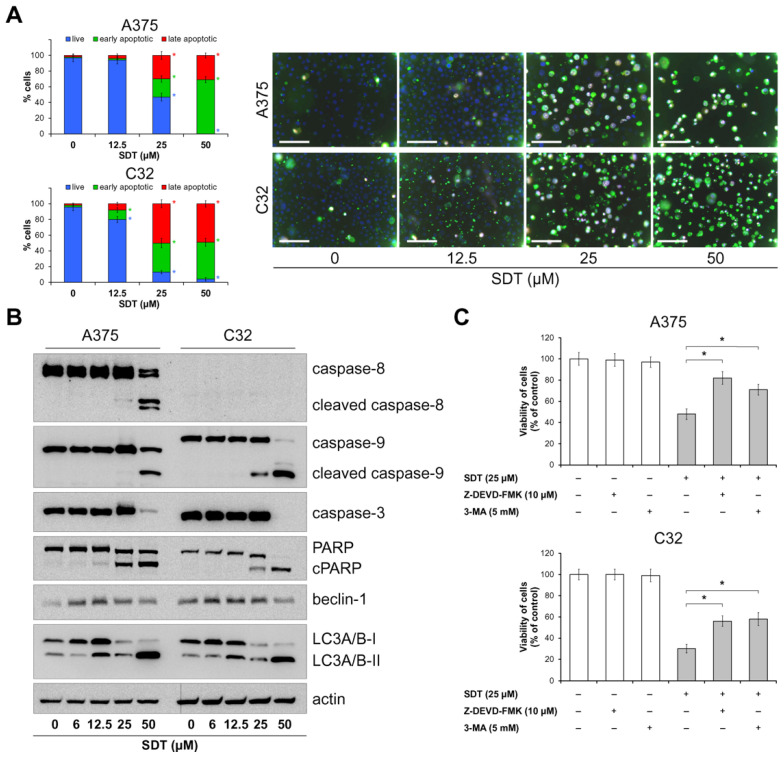
SDT promotes apoptosis and non-protective autophagy in melanoma cells. (**A**) A375 and C32 melanoma cells were treated with different concentrations (12.5–50 μM) of SDT for 48 h and stained with annexin V and propidium iodide to determine the apoptosis rate. Cell nuclei were stained with Hoechst 33342 (blue fluorescence); annexin V is green and propidium iodide is red. Scale bar 100 μm, objective ×20. (**B**) The level of apoptosis and autophagy markers in A375 and C32 cells incubated with SDT (6–50 μM) for 48 h was analyzed using Western blot. (**C**) A375 and C32 cells were pretreated with 10 μM of Z-DEVD-FMK or 5 mM of 3-methyladenine (3-MA) for 2 h and then incubated with 25 μM of SDT for 48 h. The viability of cells was determined using an MTT assay. Data are presented as mean ± SD from three independent experiments. * *p* < 0.05 vs. control unless indicated otherwise.

To determine the mechanism of cell death induced by 48 h treatment with SDT, melanoma cells were stained with annexin V and propidium iodide. The results of the assay and microphotographs of stained cells are shown in Figure 3A. The nuclei of cells were stained with Hoechst 33342 (blue). Early apoptotic cells were annexin V-positive (green fluorescence) and propidium iodide-negative, whereas late apoptotic cells were annexin V-positive and propidium iodide-positive (both green and red fluorescence). Treatment of A375 cells with 25 μM SDT resulted in an increase in the percentage of early apoptotic cells and late apoptotic cells to 23% and 30%, respectively, whereas in C32 cells these values were even higher and reached 37% and 50%. In turn, after incubation with 50 μM of SDT, almost all A375 and C32 cells were apoptotic. Based on these data, the level of proteins involved in apoptosis pathways was analyzed by Western blot. As shown in Figure 3B, treatment with SDT for 48 h led to cleavage of caspase-8, -9, and -3 in A375 cells. On the other hand, the level of caspase-8 (proenzyme) was undetectable in C32 cells; however, similarly to results obtained in C32, SDT induced cleavage of caspase-9 and -3. Notably, in both cell lines, SDT treatment led to cleavage of PARP, suggesting caspase-3 activation. In order to reveal the effect of SDT on the induction of autophagy, the level of beclin-1 and LC3A/B proteins was investigated. As shown in Figure 3B, cells treated with 6–25 μM SDT showed significantly increased levels of beclin-1. Furthermore, starting from a concentration of 12.5 μM of SDT, a marked upregulation of autophagy marker LC3A/B-II was detected. To further analyze whether inhibition in melanoma cell viability by SDT is directly related to apoptosis or autophagy, cells were pretreated with caspase-3 inhibitor Z-DEVD-FMK or autophagy inhibitor 3-methyladenine (3-MA) prior to SDT exposure. As shown in Figure 3C, the viability of cells was significantly reduced after incubation with 25 μM of SDT for 48 h. However, both inhibitors partially counteracted the cytotoxic effect of SDT in both cell lines. These findings suggest that SDT exposure triggers a caspase cascade involving intrinsic and extrinsic pathways of apoptosis and induces autophagy in melanoma cells. Inhibition of cell viability is related to both the promotion of apoptosis and initiation of autophagy.

### 2.3. SDT Inhibits Melanoma Cell Migration

**Figure 4:**
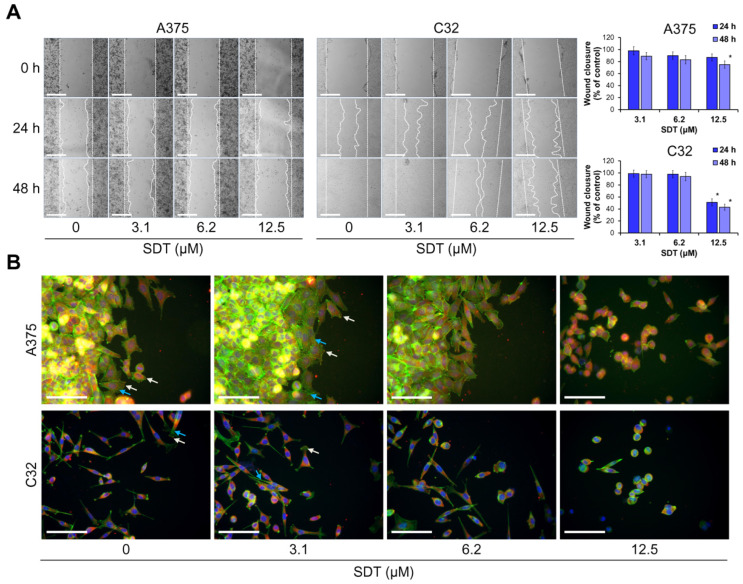
SDT affects the migration ability of melanoma cells. (**A**) A375 and C32 melanoma cell monolayers were scratched and cells were treated with different concentrations (3.1–12.5 μM) of SDT for 48 h. Representative photographs of the wound at time points 0, 24, and 48 h are shown. Scale bar 500 μm, objective ×4. Data are presented as mean ± SD from three independent experiments. * *p* < 0.05 vs. control. (**B**) Immunostaining of focal adhesion kinase (FAK) in A375 and C32 cells at the margin of the wound after incubation with different concentrations (3.1–12.5 μM) of SDT for 48 h. FAK is stained red, and white arrows point to high FAK density in focal adhesions at the cell periphery. Actin fibers are stained with phalloidin (green fluorescence) and blue arrows point to large actin bundles. Cell nuclei are stained with Hoechst 33342 (blue fluorescence). Scale bar 100 μm, objective ×20.

A wound-healing assay was used to elucidate the effect of SDT on melanoma cell migration. It was found that treatment with 12.5 μM of SDT for 48 h significantly inhibited the wound healing rate in both A375 and C32 cells (Figure 4A). Notably, used cell lines significantly differed in their ability to migrate. A375 cells exhibit epithelial morphology and low motility, whereas C32 cells express high migratory behavior. Nevertheless, incubation with SDT resulted in markedly lower migration, which coincided with the decreased recruitment of focal adhesion kinase (FAK) to the leading edge of migrating cells as determined by immunofluorescence microscopy (Figure 4B). The reduced level of FAK in focal adhesions was accompanied by a decrease in the formation of actin bundles including stress fibers. Together, these findings indicate that in non-toxic or slightly toxic concentrations, SDT suppresses cell migration by focal adhesions disassembly and depolymerization of actin bundles.

## 3. Discussion

**Figure 5:**
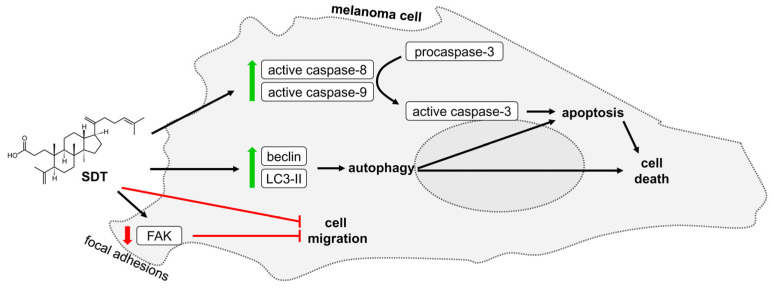
Schematic diagram illustrating the effects of SDT on melanoma cells.

Birch trees are plants of great pharmacological potential. Their parts, mainly leaves and bark, have been used for centuries in traditional medicine [16]. The chemical composition of birch buds has been studied for years and this research was intensified after the cytotoxic activity of the bud extracts was demonstrated in numerous cell lines [19,20,27,28]. Consequently, these investigations led to the isolation of flavonoids, in particular santin and cirsimaritin, that promoted apoptosis in digestive system cancer cells, endometrial adenocarcinoma, and cervical cancer, but have low effect on normal cells [29,30]. Another group of active extract components were ursane-type and oleanane-type 3,4-*seco*-triterpenoids that triggered apoptosis in gastric adenocarcinoma and colon cancer [24]. However, the role of 3,4-*seco*-triterpenoids in inducing regulated cell death of melanoma is unclear. In this study, 3,4-*seco*-dammara-4(29),20(21),24(25)-trien-3-oic acid (SDT) was isolated from buds of silver birch. Its cytotoxic activity was determined in melanoma cells, normal skin fibroblasts, and keratinocytes, and then apoptosis and autophagy were shown to contribute to SDT-induced cell death (Figure 5).

SDT is characterized by a relatively simple structure containing the carboxyl group encompassing a C3 carbon and the lipophilic large fragment containing no additional polar groups. The cytotoxicity of SDT was evaluated in melanoma cell lines (C32 and A375) and in normal skin cells. We found that treatment with SDT induced cell death in time- and concentration-dependent manners. The viability of fibroblasts was however slightly less affected compared with melanoma cells. On the other hand, there were no differences between the cytotoxicity of SDT in keratinocytes and melanoma cells. IC_50_ values of this compound in melanoma cells were 23 μM and 19 μM in A375 and C32 cells, respectively, after treatment for 48 h. There are only a few reports focused on the cytotoxicity of other 3,4-*seco*-dammaranes, but the selectivity for cancer cells was not investigated and results for normal cells were not included in these studies. Dammarenolic acid from *Aglaia sp*. (*Meliaceae*), differing from SDT by the presence of a hydroxyl group at C20, reduced the viability of the HeLa-based P4CCR5 indicator cell line with an IC_50_ value of about 20 μM after 48 h of incubation [25]. Other tested compounds contained more hydroxyl groups and an altered position of the double bond in the alkyl group at C17. For instance, a study on cyclocariols isolated from leaves of *Cyclocarya paliurus* (*Juglandaceae*) containing 3 hydroxyl groups (one bonded to C20) showed that free acids decreased viability of liver and breast cancer cells with IC_50_ values of 17–37 μM after 48 h. Interestingly, the esterification of the carboxyl group enhanced this effect by 11–40%. Particularly sensitive to the action of these esters were HCT-116 colon cancer cells with an IC_50_ value of about 5 μM [11]. Qingqianliusu G, another free acid from *C. paliurus* containing two hydroxyl groups (one bonded to C20), reduced the survival of gastric cancer, breast cancer, colon cancer, and hepatocellular carcinoma cells with IC_50_ values of 8, >20, >20, and >20 μM, respectively. However, its ethyl ester, containing an additional hydroxyl group at C12, showed significantly stronger effects with IC_50_ values for the whole cell line panel in the range of 9–13 μM [31]. These results may indicate the esterification of the carboxyl group as a direction for further research to enhance the cytotoxic effect of *seco*-triterpenoids, including SDT. The negative effect of the carboxyl group on the cytotoxic activity of the 3,4-*seco*-dammaranes can be considered a general rule since its blockade by conjugation with amino acids or its reduction to alcohol or aldehyde increased cytotoxicity up to tenfold in the 6-thioguanine resistant melanoma C32TG cells [26]. It is worth noting that 3,4-*seco*-triterpenoids belonging to ursane and oleanane types were previously isolated by us and the results showed much weaker cytotoxic effects in A375 cells than SDT. Their IC_50_ values were 101–111 μM after 48 h [24]. Thus, our study may indicate a higher potential of 3,4-*seco*-dammarane derivatives for further studies on melanoma.

The decrease in viability of SDT-treated cells may be due to stimulation of one or more types of regulated cell death, which is a desirable mechanism for potential anticancer agents [32]. Although most of these modes were reported to be induced by triterpenoids, apoptosis seems to be most common and is described frequently for both triterpenoids and *seco*-triterpenoids [24,33]. Consistently with these predictions, SDT exposure resulted in massive apoptosis of A375 and C32 cells. Two main apoptotic pathways may be engaged in SDT-induced cell death. The intrinsic pathway is induced by proteins released from the mitochondria, while the extrinsic pathway is initiated by the signal generated by death receptors; however, molecules of one pathway may influence the other. Western blot data revealed induction of caspase-9 (a component of the intrinsic pathway) in both cell lines and caspase-8 (a component of the extrinsic pathway) in A375 cells since the procaspase-8 level was below the limit of detection in the C32 cell line, as well as activation of caspase-3 (executioner caspase). Notably, the protein band for caspase-9 in C32 cells was located substantially higher on the nitrocellulose compared with A375 but cleaved caspase-9 had equal protein mass in both cell lines. This may suggest the elongation of the protein chain located outside catalytic domains or its post-translational modification in C32 cells. The nature of this phenomenon in melanoma and its influence on apoptosis requires further studies. Other reports on the effect of dammaranes on apoptosis pathways involve only compounds with an unbroken bond between C3 and C4. Treatment of prostate cancer LNCaP with 50 μM of 20(S)-25-methoxyl-dammarane-3β,12β,20-triol for 24 h led to activation of caspase-8 and caspase-9 [34]. Another dammaranes, cabralealactone 3-acetate and its methyl ether, which contain a tetrahydrofurane substituent replacing the hydrocarbon chain at C17, increased the level of death receptors DR4 and DR5, leading to activation of caspase-8 in breast cancer cell line MCF7 [35]. Similarly to SDT, 3,4-*seco*-ursanes and 3,4-*seco*-oleanane isolated by us from downy birch buds also activated both apoptosis pathways [24]. Using caspase-3 inhibitor proved that cell death is a result of apoptosis in melanoma cells exposed to SDT. The cytotoxicity assay revealed that the reduction in cell viability by SDT was partly prevented by the caspase-3 inhibitor, suggesting both that the reduction in cell viability resulted from activation of apoptosis and that another mechanism of cell death is triggered by SDT. 

Although our research is preliminary in nature, more advanced studies on 20(S)-25-methoxyl-dammarane-3b,12b,20-triol confirmed p53-dependent apoptosis leading to downregulation of Bcl-2 level, incorporation of Bax into the mitochondrial outer membrane, and activation of the intrinsic pathway [34]. Interestingly, according to The TP53 Database (R20, July 2019: https://tp53.cancer.gov, accessed on 18 August 2024) [36], both cell lines A375 and C32 express wild-type p53. Therefore, the involvement of p53 in the apoptosis induced by SDT cannot be excluded. Other molecular targets for two *seco*-dammaranes, dammarenolic acid and (12β,20S)-12,20-dihydroxy-3,4-*seco*-dammaran-4,24-dien-3-oic acid, were identified. Both compounds inhibited the tyrosine kinase activity of the epidermal growth factor receptor and insulin receptor [37]. These receptors are well-known activators of the phosphatidylinositol 3’-kinase/protein kinase B (PI3K/Akt) pathway, which control cellular metabolism, attenuate apoptosis, and promote cell survival [38]. Inhibition of Akt by other triterpenoids was also reported in numerous cancer cells [39]. Whether any of these mechanisms are involved in SDT-induced apoptosis requires further study.

Autophagy (macroautophagy) is a tightly regulated process of degradation of cellular constituents for recycling their components in eukaryotic cells. During autophagy, portions of cytoplasm-containing organelles are sequestered within double-membrane vesicles known as autophagosomes, which then undergo fusion with lysosomes [40]. Autophagosome formation is associated with the conjugation of protein LC3-I with phosphatidylethanolamine, which generates LC3-II and this product is subsequently targeted to the membrane [41]. The lipidated form of LC3 migrates faster in polyacrylamide gels during electrophoresis, making it detectable by Western blot. Treatment of melanoma cells with SDT resulted in a substantial increase in LC3-II with a concomitant decrease in LC3-I level, suggesting autophagosome formation. Autophagy is involved in maintaining homeostasis and allows the survival of cells under starvation. Therefore, inhibition of autophagy may become a new strategy to improve the efficacy of conventional chemotherapy [42]. On the other hand, in certain conditions, autophagy leads to cell death either by autophagy itself or by triggering a variety of cell death modes, for instance, apoptosis, necroptosis, and ferroptosis [43]. Importantly, structurally different types of triterpenoids were reported to induce autophagy in cancer cells [44]. Among triterpenoids contained in birch trees, the most recognized are lupanes. Betulinic acid was reported to induce the formation of autophagosomes and to upregulate LC3-II level; however, it inhibited autophagy flux. Moreover, the first steps of autophagy activated by betulinic acid were rather protective in their nature [45]. In contrast, betulin promoted autophagy-mediated apoptosis. Autophagy inhibitor chloroquine, which increases the pH of lysosome and blocks the fusion of the autophagosome with the lysosome, efficiently reverses betulin-induced reduction in the viability of osteosarcoma cells and suppresses caspase-3 cleavage [46]. A study on lupeol indicated that the upregulation of autophagy was initiated by the Akt/mTOR pathway leading to the upregulation of beclin-1, a key initiator of autophagy [47]. However, a limited number of reports on the effect of dammarane-type triterpenoids on autophagy are available and most of them are focused on triterpenoid saponines (glycosides) from ginseng (*Panax ginseng*) [48]. Thus, the effect of *seco*-dammaranes, and more generally *seco*-triterpenoids, on autophagy is currently poorly understood. The present study demonstrated that SDT upregulates beclin-1 level and it may suggest that the molecular target of the compound is located in upstream signaling pathways. Although a decrease in beclin-1 was noted at the highest SDT concentrations, these cells were also characterized by a high rate of apoptosis, and beclin-1 was reported to be degraded by the executioner caspases [49]. Autophagy inhibitor, 3-MA, partially counteracted SDT-induced reduction in cell viability. Thus, both autophagy and apoptosis contributed to melanoma cell death.

The formation of focal adhesions that provide the link between actin cytoskeleton and extracellular matrix proteins is essential for cell adhesion and migration [50]. Focal adhesion kinase (FAK) is located in focal adhesions and plays a pivotal role in the transduction of signals generated by integrins. Hence, immunostaining of FAK was used for the identification of focal adhesions in the population of migrating cells. Treatment with SDT decreased both the number of FAK-expressing puncta on the cell edge and melanoma cell migration capacity. Whether loss of adhesion also affected apoptosis in SDT-treated cells is currently not known but appears unlikely. Although FAK activates RAF/MEK/ERK mitogen-activated protein kinase (MAPK) signaling cascade to promote cell survival, in half of melanomas, including A375 and C32 cells, the function of FAK in triggering ERK is attenuated due to the activating BRAF V600E mutation [51,52]. Therefore, these cells show constitutively active MAPK pathways, thus not depending on exogenous signals.

In conclusion, our study provides evidence that SDT reduces melanoma cell viability by inducing apoptosis- and autophagy-mediated cell death. Our findings indicate 3,4-*seco*-dammaranes as a promising group of triterpenoids with anticancer activity.

## 4. Materials and Methods

### 4.1. Isolation of Triterpene Acid

Silver birch buds (300 g) were ground and extracted by carbon dioxide SFE on a Waters SFE-1000F-2-FMC50 (Milford, MA, USA) system at 50 °C and a pressure of 300 bar. The SFE extract (20 g) was separated on a silica gel column and eluted with *n*-hexane, a mixture of hexane and ethyl acetate in different volume proportions: 100:2, 100:4, 100:6, 100:8, 100:10, 100:12, and 1:1 (*v*/*v*), and ethyl acetate. Fraction (2.561 g) eluted by hexane:ethyl acetate (100:8), which was rich in a triterpene compound, was subjected to further separation using an apparatus for medium-pressure liquid chromatography (MPLC), Teledyne ISCO CombiFlash EZ Prep (Lincoln, NE, USA). Separation was performed on a RediSep Rf Gold silica gel column (Teledyne ISCO) in a gradient mode from 6 to 8% ethyl acetate in *n*-hexane. The fractions separated by MPLC were analyzed by HPLC with a diode array detector (DAD) using a scan in the range of 200–800 nm and detection at 254 and 365 nm. After multistep chromatographic separations, 75.9 mg of pure (>99% according to GC-MS data) 3,4-*seco*-dammara-4(29),20(21),24(25)-trien-3-oic acid was isolated.

The composition of the collected fractions, as well as the purity of the isolated *seco*-acid, were monitored by gas chromatography-mass spectrometry (GC-MS). The mass spectrum of the trimethylsilyl derivative of the obtained acid is shown in Figure S1. The structure of the isolated *seco*-acid was established based on ^1^H and ^13^C NMR spectra. Figure S2 gives a ^1^H NMR spectrum of the acid registered on a Bruker Avance II 400 spectrometer (Bruker, Billerica, MA, USA). The ^13^C NMR spectrum is presented in Figure S3. The results of NMR identification are in agreement with previously published data for this compound [19]. 

### 4.2. Cell Culture and Treatment

Melanoma cells A375 were obtained from Sigma-Aldrich (St. Louis, MO, USA). Melanoma cells C32 and normal human skin fibroblasts CCD-25Sk were purchased from the American Type Culture Collection. HaCaT keratinocytes were obtained from AddexBio (San Diego, CA, USA). Cells were cultured in Dulbecco’s Modified Eagle Medium (DMEM, Gibco, Waltham, MA, USA), containing 10% (*v*/*v*) fetal bovine serum (FBS, Gibco) and 1% (*v*/*v*) penicillin/streptomycin solution (Gibco) in a 5% CO_2_ incubator at 37 °C. 

SDT was dissolved in dimethyl sulfoxide (DMSO) to obtain a concentration of 100 mM. This solution was stored at −20 °C. The working solutions (1 mM) were obtained by diluting the 100 mM solution with a culture medium. The final concentration of DMSO in the culture medium never exceeded 0.1%, and 0.1% DMSO (*v*/*v*) was used as a control. Cisplatin (Sigma-Aldrich) was dissolved in a culture medium just before it was added to the cells.

### 4.3. Cell Viability Assay 

Cells were seeded in 96-well plates at 1 × 10^4^ cells per well and allowed to adhere overnight. SDT at concentrations 6.2, 12.5, 25, 50, and 100 μM was added to the culture medium, and the cells were treated for 24, 48, or 72 h. Subsequently, 3-(4,5-dimethyl-2-thiazolyl)-2,5-diphenyl-2H-tetrazolium bromide (MTT) solution was added to each well, and cells were incubated for 4 h at 37 °C. The medium was then removed and formazan crystals were dissolved in 100 μL of DMSO and 12.5 μL of Sorensen’s glycine buffer on a plate shaker. The absorbance at a wavelength of 570 nm was measured using a microplate reader. The half-maximal inhibitory concentration (IC_50_) values were calculated using GraphPad Prism v7.04 software.

### 4.4. Clonogenic Assay

In total, 250 viable melanoma cells were plated in each well of 12-well plates and allowed to adhere for 24 h. The cells were treated with various concentrations (25, 50, and 100 μM) of SDT or vehicle for 48 h. Subsequently, the SDT solution was discarded, and fresh culture medium was added to the wells. After seven days, the medium was removed and the cells were washed once with phosphate-buffered saline (PBS). Then, the cells were fixed with 4% (*v*/*v*) formaldehyde and stained with 0.1% (*w*/*v*) crystal violet. The cells were washed repeatedly with PBS to remove excess dye. Subsequently, plates were dried in the air, and photographs of wells were taken using an imager. Stained cells were solubilized in 10% acetic acid and optical density was measured at 590 nm.

### 4.5. Apoptosis Assay

Melanoma cells were seeded at a density of 1 × 10^5^ cells per well in 6-well plates. On the following day, cells were treated with SDT at concentrations of 12.5, 25, and 50 μM for 48 h. Floating and adherent cells were collected and assayed using an annexin V/propidium iodide (PI) kit (#V13242, Thermo Fisher Scientific, Waltham, MA, USA), according to the manufacturer’s instructions. Briefly, cells were resuspended in 100 μL of annexin-binding buffer containing 5 μL of annexin V–FITC conjugate solution, 1 μg/mL PI, and 1 μg/mL Hoechst 33,342 for 15 min at room temperature. Then, 400 μL of annexin-binding buffer was added, and the cell suspension was transferred to 96-well plates and visualized using a fluorescence microscope (BD Pathway 855, Becton Dickinson, Franklin Lakes, NJ, USA). Early apoptotic cells showed green and blue fluorescence, while late apoptotic cells showed green, red, and blue fluorescence.

### 4.6. Western Immunoblot

Adherent and floating melanoma cells were collected and lysed in an ice-cold RIPA buffer. The protein concentration of the homogenate was determined using the Lowry assay. Proteins (20–40 μg) were electrophoretically separated on 10% or 12% SDS-PAGE gels prior to transfer to nitrocellulose membranes and immunoblotting. Membranes were blocked with 5% (*w*/*v*) skim milk for 1 h at room temperature and incubated overnight at 4 °C with the following primary antibodies: anti-caspase-8 antibody (#9746, 1:1000), anti-caspase-9 antibody (#9508, 1:1000), anti-caspase-3 antibody (#9662, 1:1000), anti-PARP antibody (#9542, 1:1000), anti-beclin-1 antibody (#3495, 1:1000), anti-LC3A/B antibody (#12741, 1:1000) purchased from Cell Signaling Technology (Danvers, MA, USA), and anti-actin antibody (#A2066, 1:2000) obtained from Sigma-Aldrich. After washing, a secondary antibody solution in 5% skim milk (anti-mouse IgG-HRP, A9044, 1:5000 or anti-rabbit IgG-HRP, A9169, 1:5000, Sigma-Aldrich) was added for 1 h at room temperature. Membranes were incubated with ECL-HRP substrate (GE Healthcare, Chicago, IL, USA) and the signal was detected using the BioSpectrum Imaging System (Ultra-Violet Products, Ltd., Cambridge, UK).

### 4.7. Wound Healing Assay

Melanoma cells were plated at a density of 1 × 10^5^ cells per well in 24-well plates. When cells covered over 90% of the plate surface, straight lines were scratched across the center of the well with a pipette tip. Then, the medium with floated cells was discarded and the wound was gently washed twice with PBS. The adherent cells were cultured in DMEM containing 2% (*v*/*v*) FBS and treated with SDT at concentrations of 3.1, 6.2, and 12.5 μM. Gap closure was continuously monitored under a light microscope (Nikon, Tokyo, Japan) and photographed at time points of 0 h, 24 h, and 48 h. The gap distances were measured using the Image J software (v1.53c) at these time points to assess cell migration ability.

### 4.8. Immunofluorescence

Melanoma cells were plated in 96-well clear bottom black plates at 5 × 10^4^ cells per well. When the cells covered over 90% of the plate surface, a straight line was scratched gently across the center of the well with a pipette tip. Then, the medium with floated cells was discarded and the wound was gently washed twice with PBS. The adherent cells were cultured in DMEM supplemented with 2% (*v*/*v*) FBS and treated with SDT at concentrations of 3.1, 6.2, and 12.5 μM for 24 h. After SDT treatment, the medium was removed and the cells were fixed for 15 min with 4% (*v*/*v*) paraformaldehyde, permeabilized for 5 min with 0.1% (*v*/*v*) Triton X-100, and blocked with 10% (*v*/*v*) heat-inactivated goat serum for 1 h at room temperature. The cells were incubated with anti-FAK antibody (sc-1688, Santa Cruz, Santa Cruz, CA, USA) overnight at 4 °C. Then, the cells were incubated with Alexa Fluor 594 conjugated antibody (A11032; Invitrogen, Waltham, MA, USA) for 1 h in the dark. Cell nuclei and F-actin were counterstained with Hoechst 33342 (Invitrogen) and Phalloidin-Atto 488 (Sigma-Aldrich), respectively. Images of migrating cells were taken with a BD Pathway 855 confocal microscope.

### 4.9. Statistical Analysis

The results were analyzed in GraphPad Prism v7.04 software using a one-way ANOVA followed by Tukey’s test; a *p*-value < 0.05 was considered statistically significant.

## 5. Conclusions

SDT, the 3,4-*seco*-dammarane containing a carboxyl group encompassing a C3 carbon and no additional polar groups, showed significant cytotoxic activity in melanoma cell lines. It induced intrinsic and extrinsic pathways of apoptosis, promoted non-protective autophagy, and attenuated cell migration. The data confirm the potential of this kind of structure in melanoma cell cultures and may indicate it as a parent structure for derivative synthesis in the future. Subsequent studies should be continued in the expanded panel of melanoma cell lines with a focus on the identification of molecular targets of SDT and confirmation in animal models.

## Data Availability

The original contributions presented in this study are included in the article and the Supplementary Materials. Further inquiries can be directed to the corresponding author.

## Supplementary Materials

The following supporting information can be downloaded at https://www.mdpi.com/article/10.3390/molecules29174091/s1: Figure S1: Mass spectrum of trimethylsilyl derivative of 3,4-*seco*-dammara-4(29),20(21),24(25)-trien-3-oic acid; Figure S2: ^1^H NMR spectrum of 3,4-*seco*-dammara-4(29),20(21),24(25)-trien-3-oic acid; Figure S3. ^13^C NMR spectrum of 3,4-seco-dammara-4(29),20(21),24(25)-trien-3-oic acid.
