# Supplementary material for: Dammarane-Type 3,4-seco-Triterpenoid from Silver Birch (Betula pendula Roth) Buds Induces Melanoma Cell Death by Promotion of Apoptosis and Autophagy
Source: Molecules. 2024 Aug 29;29(17):4091. doi: 10.3390/molecules29174091 (PMC11397366; doi:10.3390/molecules29174091)
Supplement: Supplementary file 1 [file molecules-29-04091-s001.zip › molecules-3132339-supplementary.pdf]

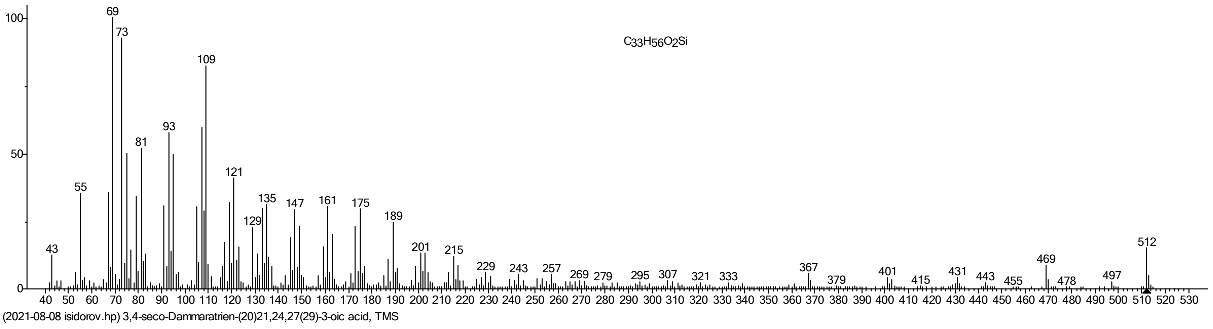

Figure S1. Mass spectrum of trimethylsilyl derivative of 3,4-*seco*-dammara-4(29),20(21),24(25)-trien-3-oic acid

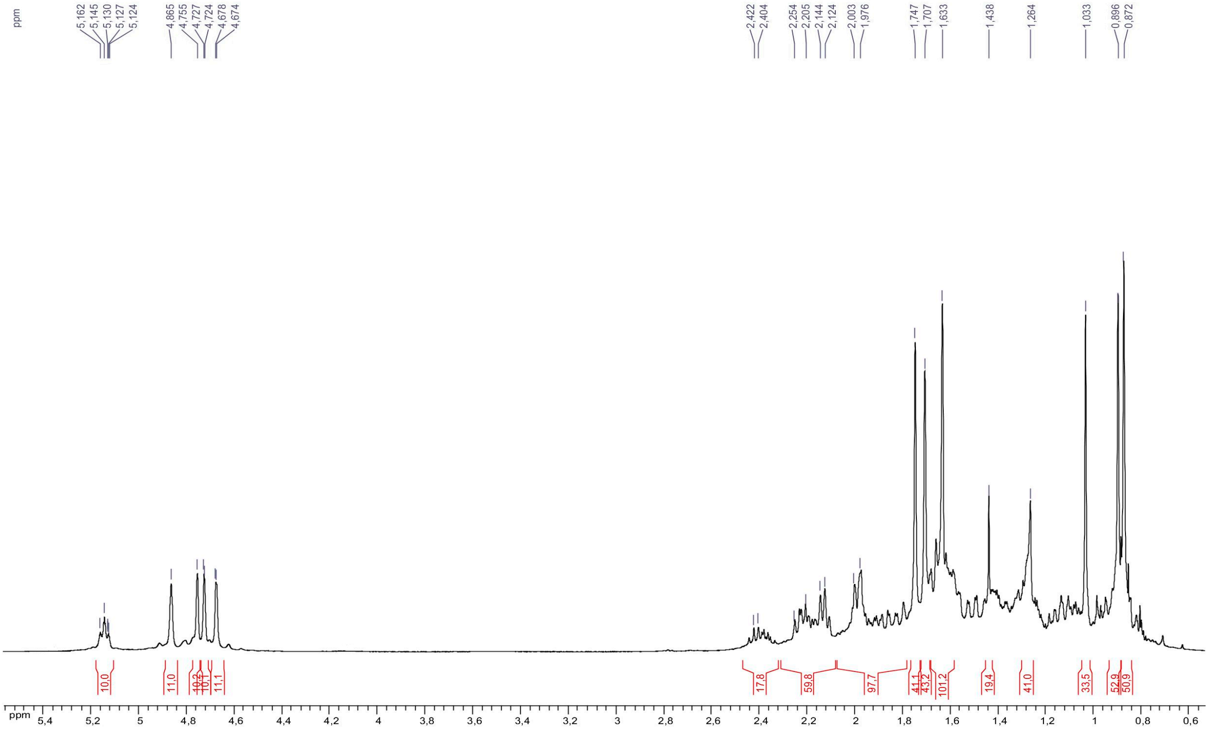

Figure S2. ^{1}H NMR spectrum of 3,4-*seco*-dammara-4(29),20(21),24(25)-trien-3-oic acid

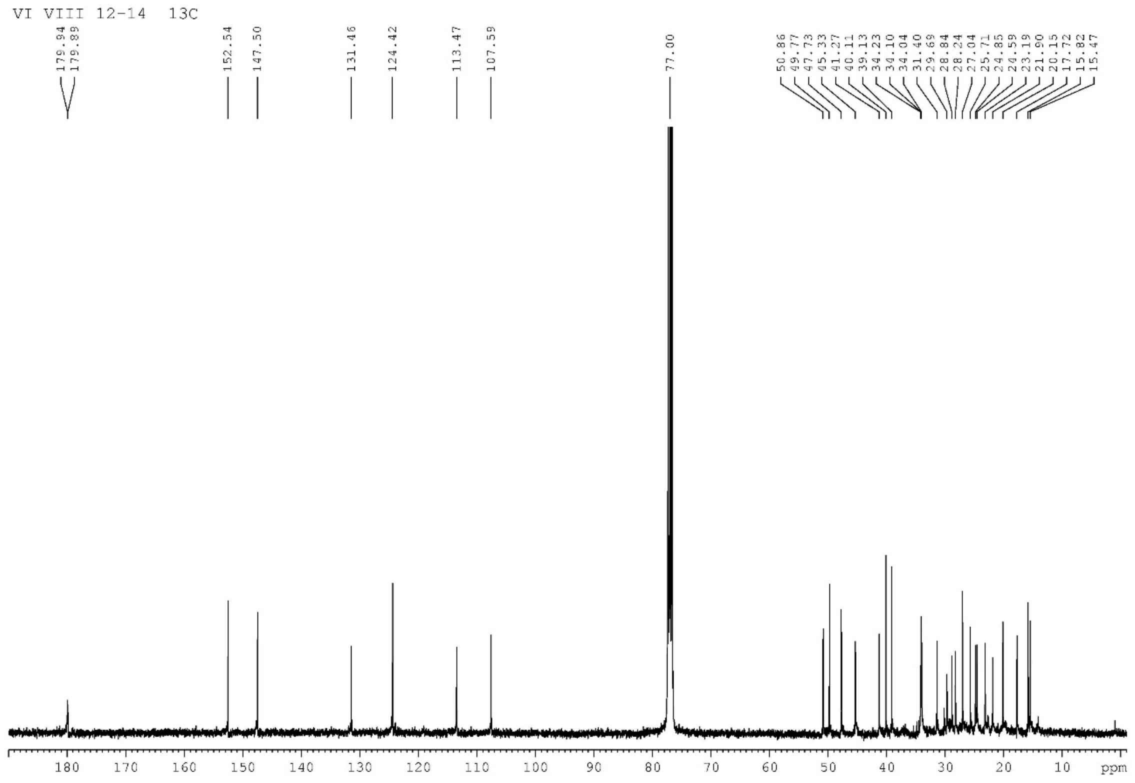

Figure S3. ^{13}C NMR spectrum of 3,4-*seco*-dammara-4(29),20(21),24(25)-trien-3-oic acid
